# Chimeric antigen receptor NK-92 cell function is modulated by HLA class I expression of target cells

**DOI:** 10.1016/j.isci.2025.112523

**Published:** 2025-04-23

**Authors:** Nicolai Stransky, Ranran Ji, Lukas Prause, Katrin Ganser, Winfried S. Wels, Peter Ruth, Stephan M. Huber, Franziska Eckert

**Affiliations:** 1 Department of Radiation Oncology, University Hospital Tübingen, 72076 Tübingen, Germany; 2 Department of Pharmacology, Toxicology and Clinical Pharmacy, Institute of Pharmacy, University of Tübingen, 72076 Tübingen, Germany; 3 Georg-Speyer-Haus, Institute for Tumor Biology and Experimental Therapy, Frankfurt, Germany; 4 Frankfurt Cancer Institute (FCI), Goethe University, Frankfurt, Germany; 5 German Cancer Consortium (DKTK), Partner Site Frankfurt/Mainz, a partnership between DKFZ and University Hospital Frankfurt, Frankfurt, Germany; 6 Department of Radiation Oncology, Medical University Vienna, AKH, Comprehensive Cancer Center, Wien, VA, Austria

**Keywords:** Biological sciences, Immunology, Components of the immune system, Specialized functions of cells

## Abstract

Chimeric antigen receptors – natural killer (CAR-NK) cells are a promising new cancer treatment approach. They possess distinct benefits over CAR-T cells, including an intrinsic ability to differentiate between malignant and non-malignant cells, and a lower risk of graft-versus-host disease. Here, we evaluated two CAR-NK-92 cell lines, targeting either CD276 or HER2, and their potential on-target/off-tumor effects *in vitro*. CD276-directed CAR-NK-92 cells showed little dependence on target antigen density, while HER2-directed CAR-NK-92 displayed a pronounced dependence on target antigen density, mostly sparing target cells with low amounts of HER2. The activity of both cell lines was modulated by the expression of HLA-I on target cells, ultimately reducing their activity against non-malignant cells. Lower modulation of activity of HER2-directed CAR-NK-92 can be explained by the lower surface expression of inhibitory NK receptors, such as NKG2A and ILT-2. These results underscore the importance of thoroughly testing new cell products to fine-tune anti-cancer activity, while limiting potential on-target/off-tumor toxicity.

## Highlights

• Both CAR-NK-92 cell clones have similar activation thresholds
• Dependence of killing activity of CAR-NK-92 cell clones on target antigen density varied
• HLA-I molecules on normal cells regulate the activity of CAR-NK-92 cells
• This modulation is driven by the expression of inhibitory receptors on CAR-NK-92 cells

## Graphical abstract

**Figure:**
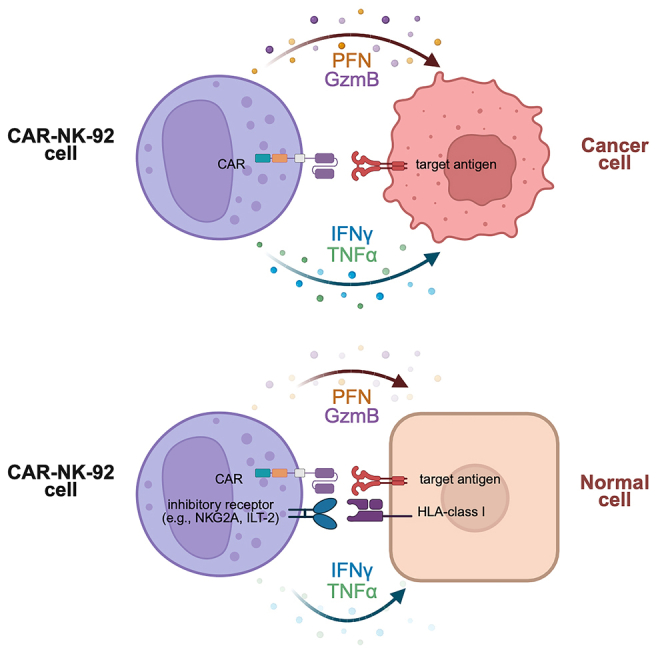

## Introduction

Chimeric antigen receptor (CAR) T cells are an important new therapeutic option in hematological malignancies.^1^ Several CAR products are approved for clinical use, which target either CD19 or the B-cell maturation antigen (BCMA). While CD19-specific CAR-T cells induce complete remission in a sizable proportion of leukemia and lymphoma patients,^2^ to date the translation of CAR-T cells to solid tumors has not yet resulted in promising efficacy data in large-scale clinical trials. The main obstacles in targeting solid tumors are thought to be the insufficient penetration of CAR-T cells into the tumor tissue, the immunosuppressive tumor microenvironment, potentially dampening the effector function of CAR-T cells, and the selection of a suitable antigen.^3^ Targeting a tumor-associated, but not tumor-specific antigen, may lead to on-target/off-tumor toxicity, i.e., CAR-T cells attacking non-cancerous cells which also express the target antigen. This has been noted for different CAR-T cell products,^4^^,^^5^ with one case report describing a fatal lung injury after the infusion of HER2-directed CAR T cells.^6^

Some of these difficulties may be ameliorated by the use of CAR-engineered natural killer (NK) cells, which theoretically can differentiate between malignant and normal cells. Mechanistically, the expression of HLA class I molecules (HLA-I) by normal tissues activates inhibitory receptors on NK cells, such as killer cell immunoglobulin-like receptors (KIRs) or NKG2A, ultimately inhibiting their effector functions.^7^ Along these lines, two reports suggested decreased CAR-NK cell activity against HLA-I-expressing normal cells. Notably, the CAR-NK cells used in these studies were derived from peripheral blood mononuclear cells or induced pluripotent stem cells.^8^^,^^9^ Additionally, results from early phase clinical trials with CAR-NK cells suggest a low risk of cytokine release syndrome or neurotoxic adverse events,^10^^,^^11^^,^^12^^,^^13^ both of which commonly occur after CAR-T cell administration.^14^ Lastly, as NK cells do not express T cell receptors, a reduced risk of graft-versus-host disease (GvHD) is expected and, so far, no GvHD has been observed even after administering HLA-mismatched CAR-NK products.^10^^,^^11^^,^^13^ This potentially allows the development of CAR-NK cells as off-the-shelf cell therapeutics. An NK cell line commonly used for the generation of CAR-NK cells is NK-92,^15^^,^^16^ which was initially established from a non-Hodgkin’s lymphoma patient.^17^ NK-92 cells proliferate rapidly *in vitro* and are a promising source for upscaling of off-the-shelf cell products.^18^ On the other hand, due to their origin from a lymphoma patient, in current clinical trials CAR NK-92 cells are irradiated with 10 Gy before infusion into patients, to avoid uncontrolled proliferation and potential induction of a secondary malignancy.^11^^,^^12^ While NK-92 cells maintain their anti-cancer functions temporarily after irradiation,^19^^,^^20^ this probably necessitates repeated administration of CAR NK-92 cells, in contrast to other CAR-NK^10^ or -T cell products.^21^^,^^22^ NK-92 cells exhibit an activated NK-cell phenotype, lacking surface expression of most inhibitory receptors, except for NKG2A, ILT-2, and KIR2DL4.^16^^,^^23^ This may affect tumor cell selectivity and, hence, increase the risk of adverse effects.^24^

CD276 (also known as B7-H3) is an immune-checkpoint with several additional pro-tumorigenic properties, such as increased migration or chemoresistance of cancer cells. As its expression is reportedly upregulated in cancer tissue,^25^ several CAR constructs targeting CD276 are already in the clinical testing phase (e.g., NCT05835687 and NCT04077866). HER2 (also known as ErbB2) is one of the most commonly selected target antigens for CAR NK cell therapy of solid cancers^15^ (e.g., NCT01109095 and NCT03383978). The HER2 receptor tyrosine kinase is also present at low levels in healthy epithelial tissues, with *HER2* gene amplification occurring in around 15–30% of breast cancers, and other mechanisms of *HER2* overexpression described for other cancer entities.^26^ Importantly, no on-target/off-tumor toxicities have been reported in clinical trials with T cells and NK-92 cells engineered with HER2-specific CARs that were based on antibody FRP5.^27^^,^^28^^,^^29^^,^^30^

Our present study aimed to evaluate the influence of CAR target antigen density and HLA-I expression by target cells on the activity of two previously established CAR NK-92 cell lines, directed either against CD276^31^ or HER2,^32^ as important determinants for these cells’ therapeutic efficacy.

## Results

### CD276-CAR NK-92 cells show little dependence on antigen density

**Figure 1:**
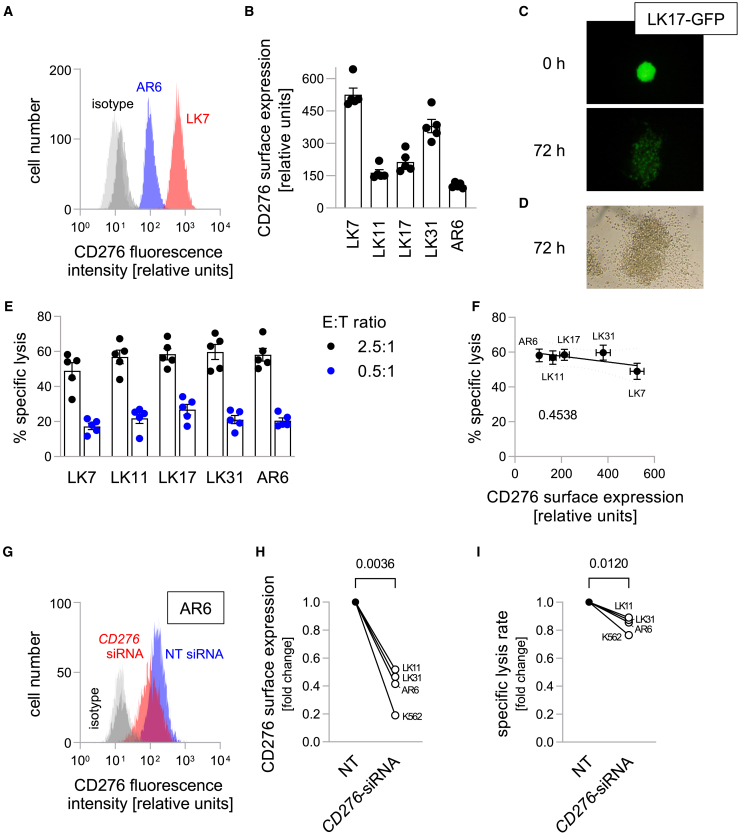
pGSCs express CD276 on their surface and are lysed by CD276-directed CAR NK-92 cells (A) Representative histograms of CD276-associated fluorescence intensity and of respective isotype controls (IC) of lowest (AR6, blue; IC: dark gray) and highest (LK7, red; IC: light gray) CD276-expressing glioblastoma cell lines. (B) Quantification of CD276 surface expression of all tested pGSCs as measured by flow cytometry. Individual data points plus superimposed means ± standard errors (SE; *n* = 5) are given. (C and D) Fluorescence (C) and brightfield (D) micrographs of GFP-transduced LK17 cells directly after the addition of CD276-directed CAR NK-92 cells (0 h, C, top) and after 72 h (C, bottom and D) indicating CAR NK-92-mediated cell lysis. (E) Quantification of cell lysis by CD276-directed CAR NK-92 cells as measured by calcein release assay for all tested pGSCs. Individual data points plus superimposed means ± SE (*n* = 5) are given for two effector/target (E:T) cell ratios. (F) Lack of a significant correlation of mean target antigen density and mean respective lysis rate (±SE). The given number indicates the goodness of fit (r2) as calculated by linear regression. (G) Representative histogram of CD276-associated fluorescence intensity and respective isotype control of AR6 cells after CD276-directed (red; IC: light gray) or non-targeting (blue; IC: dark gray) siRNA treatment. (H and I) Mean reduction in CD276 surface expression (H) and mean specific CD276-directed CAR NK-92-mediated lysis rates (E:T ratio 2.5:1; I) after CD276-downregulation normalized to the non-targeting siRNA control (n = 2–3 per cell line). Absolute values from each experiment are shown in Figure S4. The given numbers indicate error probability as *p* values calculated by paired t-test.

CD276 is reportedly overexpressed in many different cancer entities, but can also be present in healthy tissues.^25^ As such, previous research has found increased expression of CD276 mRNA and protein in glioblastoma as compared to healthy brain tissue.^33^ To confirm these findings, we screened several patient-derived stem cell-enriched glioblastoma cells (pGSCs) for their CD276 surface expression by flow cytometry. All cell lines expressed CD276, although to a varying degree (Figures 1A and 1B). As effector cells, we used a previously described CD276-directed CAR NK-92 cell line.^20^^,^^31^ While incubation with parental NK-92 did not lead to lysis of the pGSCs (Figures S1A and S1B), CD276-directed CAR NK-92 cells lysed all glioblastoma cell lines (Figures 1C, 1D; and S1A). Notably, the different levels of CD276 expressed by target cells had only little influence on the cytotoxic functions of CD276-directed CAR NK-92. To confirm previously shown CD276 specificity,^31^ we screened other cancer cell lines for their CD276 expression. We included K562 cells, with a low (approximately 10% of the surface expression of the highest expressing pGSC LK7) and Jurkat j16 cells, for which we could not detect specific CD276 expression on their surface (Figures S2A and S2B). While intrinsically NK-sensitive K562 cells were lysed to a very similar extent as the glioblastoma cells, no increase in lysis rates over parental NK-92 cells was found for Jurkat j16 cells, demonstrating CD276 specificity of CAR NK-92 cells lysis.

Irradiation of cancer cells can reportedly upregulate CD276.^33^^,^^34^ In addition, irradiation of target cells may increase NK cell activity due to the upregulation of NKG2D ligands.^35^^,^^36^ Therefore, we irradiated the pGSCs before co-incubation with the CD276-directed CAR NK-92 cells. Forty-eight hours after irradiation with 8 Gy, three out of four tested cell lines showed a slight to moderate upregulation of CD276. However, irradiation did not meaningfully affect respective lysis rates as compared to non-irradiated target cells (Figures S3A–S3G).

To further probe the influence of target antigen density, we performed knockdown experiments with siRNA directed against *CD276* mRNA, resulting in reductions in CD276 surface expression of 50–90% (Figures 1G and 1H). The lysis rates were consistently reduced after anti-*CD276* siRNA as compared to non-targeting control siRNA (Figure 1I, *p* < 0.001; Figure S4), suggesting some dependence on target antigen density of the CD276-directed CAR NK-92 activity. However, the observed reductions in cell killing activity were rather small (5–27%). Even after pronounced downregulation of the target antigen in K562 cells (with the lowest basal CD276 surface expression), the CD276-directed CAR NK-92 cells maintained most of their activity (Figures 1I, S4E and S4F). Overall, these results point toward an all-or-nothing effect of CD276 target antigen density on CD276-directed CAR NK-92 cell activity rather than a gradual dependence.

### CD276-CAR NK-92 cells spare HLA-expressing non-malignant cell lines

**Figure 2:**
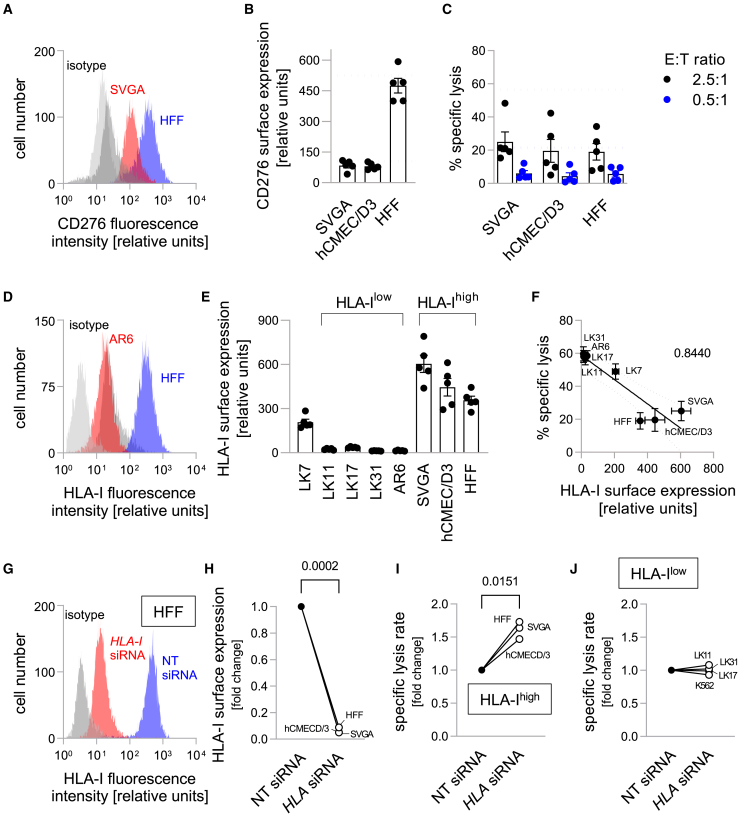
HLA-I expression of target cells modulates cytotoxicity of CD276-directed CAR NK-92 cells (A) Representative histograms of CD276-associated fluorescence intensity and of respective IgG isotype controls of SVGA (red and light gray) and HFF (blue and dark gray) cells. (B) CD276 surface expression of the tested non-transformed (normal) cell lines as measured by flow cytometry. Dotted lines indicate mean CD276 surface expression of lowest-expressing AR6 and highest expressing LK7 glioblastoma cells. (C) Cell lysis by CD276-directed CAR NK-92 cells as measured by calcein release assay with non-transformed, normal target cells. Dotted lines again depict mean minimal and maximal values observed with pGSCs. (D) Representative histograms of HLA-I-associated fluorescence intensity and respective IgG isotype controls of AR6 (red and light gray) and HFF (blue and dark gray) cells. (E) HLA-I surface expression of all tested cell lines as measured by flow cytometry. Data in (b, c, e) are represented as individual values with superimposed means ± SE (*n* = 5). (F) Relationship between mean HLA-I surface expression and mean lysis rate (±SE) indicating an inverse correlation. The given number indicates goodness of fit (r2) as calculated by linear regression. (G) Representative histograms of HLA-I-associated fluorescence intensity and respective IgG isotype controls for HFF cells after HLA-I-directed (red and light gray) or non-targeting (blue and dark gray) siRNA treatment. (H–J) Downregulation of mean (n = 2–3 experiments per cell line) HLA-I surface expression (H) leads to increased mean (n = 2–3 experiments per cell line) specific CD276-directed CAR NK-92-mediated (E:T ratio 2.5:1) lysis of HLA-I^high^ cell lines (I), but not of HLA-I^low^ cell lines (J) as normalized to values obtained for cells modified with the non-targeting siRNA control. Absolute specific lysis rates are given for every cell line in Figure S6. Numbers given in (H and I) indicate *p* values as calculated by paired t-test.

These findings led us to further analyze CD276-directed CAR NK-92 effects against non-neoplastically transformed, immortalized cell lines and fibroblasts, for which low, but still detectable CD276 expression has been reported.^25^ We included SVGA, immortalized cells of astroglial origin, hCMEC/D3, immortalized brain endothelial cells, as well as human foreskin fibroblasts (HFFs) in our further experiments. In flow cytometric analyses, all three cell lines showed specific CD276 surface expression (Figures 2A and 2B). While CD276 surface expression of SVGA and hCMEC/D3 cells was in the range of lower-expressing pGSCs (and K562 cells), HFFs showed a more pronounced CD276 surface expression, which was comparable to the highest-expressing pGSC (LK7). Nevertheless, specific lysis rates for these non-malignant targets after co-incubation with CD276-directed CAR NK-92 cells were less than half of those observed with the cancer cells (Figure 2C). We additionally performed a flow cytometry based killing assay to analyze the effect of longer coincubation periods. Among the tested cell lines, cancer cell lines LK11 and K562 were lysed to a similar extent as the normal cell line SVGA. On the other hand, HFFs were only lysed to a small extent, underscoring the previous results (Figure S5).

We hypothesized that CAR NK-92 cell activity may be modulated by the HLA-I expression of target cells, as is expected for CAR-independent natural cytotoxicity of NK cells.^7^ Indeed, while all tumor cell lines but LK7 expressed HLA-I only to a negligible extent, all three tested cell lines of non-malignant origin showed a strong signal of HLA-I surface expression (Figures 2D, 2E and S2D). HLA-I surface expression of target cells correlated negatively with respective cell lysis (Figure 2F), which can explain the different lysis rates observed for cancer and non-malignant cell lines with comparable CD276 expression. Next, we downregulated HLA-I surface expression by siRNA knockdown directed against β2 microglobulin (*B2M;* henceforth termed anti-*HLA-I* siRNA). B2M is a subunit of HLA-I molecules, necessary for their functional surface expression.^37^ The average downregulation efficacies were around 94% in HLA-I^high^ non-malignant cell lines (Figures 2G and 2H). This led on average to a 63% increase in cell lysis rates by CD276-directed CAR NK-92 cells (Figure 2I), compared to no changes for anti-*HLA-I* siRNA treated HLA-I^low^ cells (all cancer cells, except for LK7; Figures 2J and S6). These data indeed demonstrate a modulation of CAR NK-92 cell activity by the HLA-I expression of target cells.

**Figure 3:**
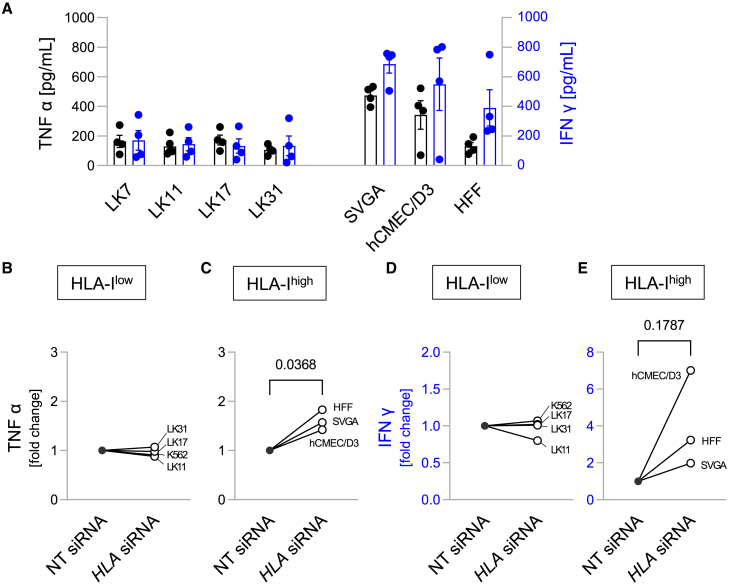
HLA-I surface expression of target cells modulates secretion of TNFα and IFNγ by CD276-specific CAR NK-92 cells (A) Secretion of TNFα (left y axis, black) and IFNγ (right y axis, blue) after co-incubation of CD276-directed CAR NK-92 cells with the indicated target cell lines as measured by ELISA (E:T ratio 1:1; given are individual data points plus superimposed means ± SE; *n* = 4). (B–E) HLA-I-directed siRNA treatment increases TNFα and IFNγ secretion for target cells expressing high levels of HLA-I, whereas no changes are observed for siRNA-treated cell lines with low levels of HLA-I. Values (means from n = 2–3 experiments per cell line) were normalized to those obtained for cells treated with non-targeting siRNA control. Absolute cytokine levels are given in Figure S7. Numbers indicate *p* values as calculated by paired t-test.

To account for additional effector functions of CAR-NK cells besides direct tumor cell killing, we tested TNFα and IFNγ secretion of CD276-directed CAR NK-92 cells after co-incubation with all target cell lines tested before in cell killing experiments (except AR6; Figure 3A and S2F). Interestingly, cytokine secretion by CD276-targeted CAR NK-92 cells was more pronounced after co-incubation with non-malignant cell lines and K562 cells, whereas all glioblastoma cells only led to moderate TNFα and IFNγ secretion. No production of TNFα and IFNγ was observed after co-incubation of the CAR-NK cells with CD276-negative Jurkat j16 cells, confirming CD276 specificity of the CD276-directed CAR NK-92 cells (Figure S2E). For HLA-I^high^ target cells, HLA-I knockdown by siRNA led to an increase of TNFα (*p* = 0.0368, Figure 3C) and IFNγ (*p* = 0.1787, Figure 3E) secretion by the CAR-NK cells, although for IFNγ the extent of the increase varied considerably between the three target cell lines. As expected, siRNA-mediated knockdown had no effect in the case of initially HLA-I^low^ target cell lines (Figures 3B and 3D).

### HER2-CAR NK-92 show a profound dependency on target antigen density

**Figure 4:**
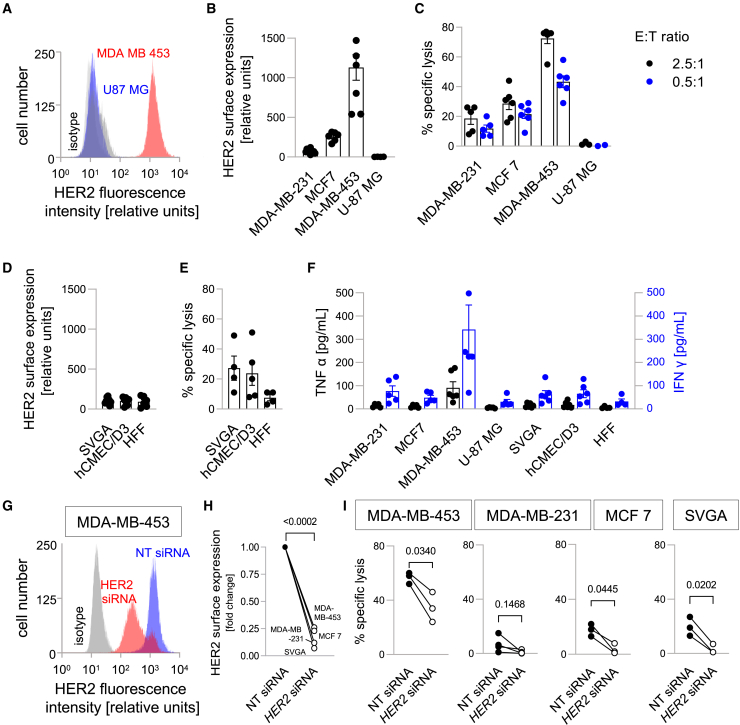
Breast cancer and non-transformed, normal cells express HER2 on their surface and are lysed by HER2-specific CAR NK-92 cells (A) Representative histograms of HER2-associated fluorescence intensity and respective IgG isotype controls of the lowest (U-87MG, blue and dark gray) and highest (MDA-MB-453, red and light gray) expressing tumor cell lines. (B and C) HER2 surface expression (B) and HER2-specific CAR NK-92-mediated (E:T = 2.5:1, black, and 0.5:1, blue) lysis of MDA-MB-231, MDA-MB-453, and MCF7 breast cancer and U-87MG glioblastoma cells (C) as measured by flow cytometry and calcein release assay, respectively. (D and E) HER2 surface expression (D) and HER2-specific CAR NK-92-mediated (E:T = 2.5:1) lysis of non-transformed SVGA, hCMEC/D3 and HFF cells (E) as analyzed in (B and C). (F) Secretion of TNFα (left y axis, black) and IFNγ (right y axis, blue) after co-incubation of HER2-directed CAR NK-92 cells and the indicated target cell lines as measured by ELISA (E:T ratio 1:1. Data in (b-f) represent individual data points with superimposed means ± SE (n = 4–6). (G) Representative histograms of HER2-associated fluorescence intensity and respective IgG isotype controls of MDA-MB-453 cells after HER2-directed (red and light gray) or non-targeting control (blue and dark gray) siRNA treatment. (H) Mean HER2 surface expression after HER2 siRNA treatment normalized to the respective non-targeting siRNA controls (n = 2–3 experiments per cell line). (I) Lysis rates by HER2-directed CAR NK-92 cells (E:T ratio 2.5:1) of MDA-MB-231, MDA-MB-453, MCF7 and SVGA cells treated with non-targeting or HER2-directed siRNA (*n* = 3). Numbers indicate *p* values as calculated by paired t-test.

To investigate whether these findings can be generalized for other CAR-engineered NK-92 cells, we performed similar experiments with a second previously established CAR NK-92 cell line which is directed against HER2.^19^^,^^32^ Both CAR NK-92 cell lines carry a similar second-generation CAR construct with a CD8α hinge region, CD28 transmembrane and costimulatory domains, and a CD3ζ signaling domain. The pGSCs endogenously expressed no or only negligible amounts of HER2 on their surface (Figure S8). Therefore, we screened additional breast cancer and glioma cell lines for HER2 surface expression by flow cytometry (Figures 4A and 4B). Among them, we detected strong and very strong HER2 expression by the breast cancer cell lines MCF7 and MDA-MB-453, respectively, compared to low to intermediate HER2 surface expression by MDA-MB-231 cells. U-87MG glioma cells served as a HER2-negative control. In contrast to the moderate and statistically not significant correlation between target antigen density and effector cell activation established in our experiments with CD276-directed CAR NK-92 cells, the cell killing activity of HER2-directed CAR NK-92 cells demonstrated a pronounced correlation with the HER2 expression level on target cell lines (Figures 4B and 4C). Lysis rates against cancer cell lines MDA-MB-231 and MCF7 with intermediate to high HER2 surface expression were around 20–30%. Specific lysis rates against MDA-MB-453 cells (with very high HER2 expression) were up to 70%, indicating a strong dependence of cell killing activity on target antigen density. Incubation of HER2-directed CAR NK-92 cells with HER2-negative U-87MG glioma cells did not result in cell lysis, demonstrating HER2 specificity of the observed effects.

To assess whether this dependence on HER2 expression levels is also found for non-malignant target cells, we first analyzed the three non-transformed cell lines SVGA, hCMEC/D3 and HFF for their HER2 expression. In flow cytometric analyses, all three cell lines showed HER2 expression at a low to intermediate level, which was comparable to the HER2 expression level of MDA-MB-231 cells (Figure 4D). After co-incubation with HER2-directed CAR NK-92 cells, specific lysis rates against SVGA and hCMEC/D3 cells were similar to those against MCF7 and MDA-MB-231 cells, while specific lysis rates against HFF were much lower (Figure 4E). Similarly to the findings of the cytotoxic function of HER2-specific CAR NK-92 cells, TNFα and IFNγ secretion correlated with the target antigen density (Figure 4F). While we found no to very low cytokine secretion after co-incubation with U-87MG and HFF cells, the IFNγ secretion was moderate for all cell lines except MDA-MB-453, for which we detected a strong secretion of IFNγ. In general, TNFα secretion was very low for every cell line, except for MDA-MB-453 cells, which led to a moderate secretion of TNFα after co-incubation with the HER2-specific CAR NK-92 cells.

To further explore the influence of target antigen density, we performed knockdown experiments with siRNA directed against *HER2* mRNA, leading to a reduction in HER2 surface expression on the target cells of 80–90% (Figures 4G and 4H). This also resulted in a pronounced reduction of target cell lysis by the CAR-NK cells (Figure 4I). More specifically, *HER2* knockdown in cell lines with low-to-intermediate basal HER2 levels led to a near-complete loss of cell lysis (MDA-MB-231, MCF7 and SVGA), whereas lysis rates were approximately reduced by half for siRNA-treated MDA-MB-453 cells with very high basal HER2 levels. Overall, these results demonstrate a strong dependence of the HER2-directed CAR NK-92 cell activity on target antigen density, which contrasts our results obtained with CD276-directed CAR NK-92 cells. Interestingly, exploratory experiments hinted at similar activation thresholds of both CAR NK-92 cells with secretion of IFNγ and TNFα beginning at 0.16–0.4 nmol/L HER2 protein for HER2-specific CAR NK-92 cells and 0.4–1.0 nmol/L CD276 protein for CD276-specific CAR NK-92 cells (Figure S9).

### HLA expression reduces activity of HER2-CAR NK-92 cells only to a small account due to a decreased expression of inhibitory surface receptors

**Figure 5:**
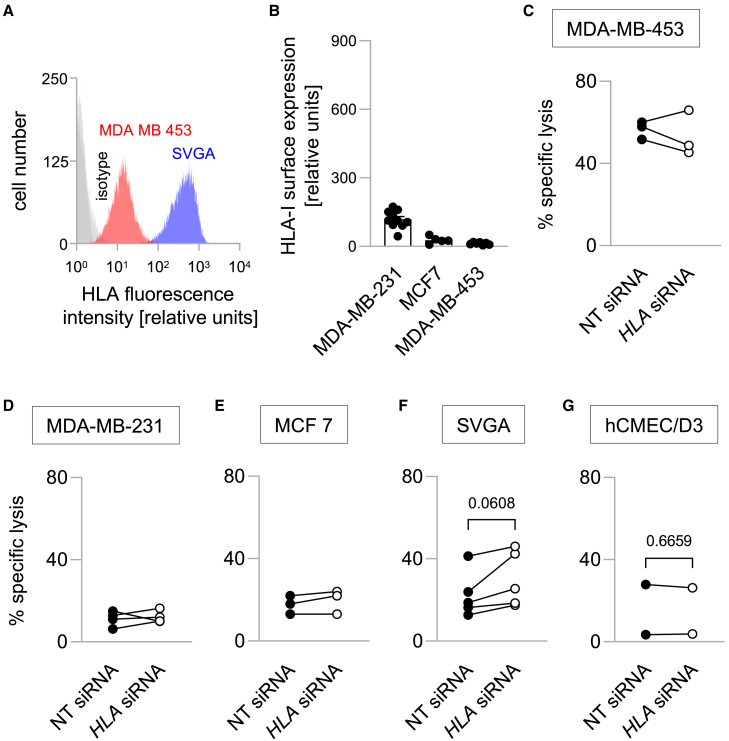
HLA-I expression of target cells has a moderate influence on the cytotoxicity of HER2-specific CAR NK-92 cells (A) Representative histograms of HLA-I-associated fluorescence intensity, and respective IgG isotype controls of low (MDA-MB-453, red and light gray) and high (SVGA, blue and dark gray) expressing cell lines. (B) HLA-I surface expression of MDA-MB-231, MCF7 and MDA-MB-453 cells as measured by flow cytometry. (C–G) Downregulation of HLA-I by siRNA does not change mean specific lysis mediated by HER2-directed CAR NK-92 cell (E:T ratio 2.5:1). Only SVGA cells displayed a numerical increase in the specific lysis rate (n = 2–5). Numbers indicate *p* values as calculated by paired t-test.

Since lysis of the non-malignant cell lines SVGA and hCMEC/D3 was similar to that of MDA-MB-231 and MCF-7 breast cancer cells with comparable HER2 expression, we investigated whether other factors, such as HLA-I expression, may still modulate HER2-directed CAR NK-92 cell activity. The tested tumor cell lines displayed negligible HLA-I expression in flow cytometry analyses, except for MDA-MB-231 cells that showed an intermediate level of HLA-I expression (Figures 5A and 5B). To explore the possible influence of HLA-I expression by target cells on HER2-directed CAR NK-92 cell cytotoxicity further, we treated the cells with anti-*HLA-I* siRNA. *HLA-I* siRNA led to no discernible effects on the lysis rates against HLA-I^low^ or HLA-I^intermediate^ cancer cell lines (Figures 5C–5E). Interestingly, in the case of non-malignant target cell lines, we found a consistent increase of lysis rates against siRNA-treated SVGA cells (Figure 5F, *p* = 0.0608), whereas no changes were seen with hCMEC/D3 cells (Figure 5G). Generally, these results imply that the activity of HER2-directed CAR NK-92 cells is modulated in a less stringent manner by HLA-I expression of target cells than that of CD276-directed CAR NK-92 cells. This may be due to differences in the surface expression of inhibitory receptors between the two CAR NK-92 cell lines. Indeed, we found decreased surface expression of both ILT-2 and NKG2A in HER2-CAR as compared to CD276-CAR NK-92 cells with only low levels of other inhibitory (e.g., KIR2DL1 and KIR2DL4) or activating receptors (NKp30, NKG2D) (Figure S10). Prior irradiation of effector cells with 10 Gy had no effect on CD276-CAR NK-92 cells and led to small increases of the expression of NKG2A in HER2-CAR NK-92 cells after 48 h (Figure S11).

**Figure 6:**
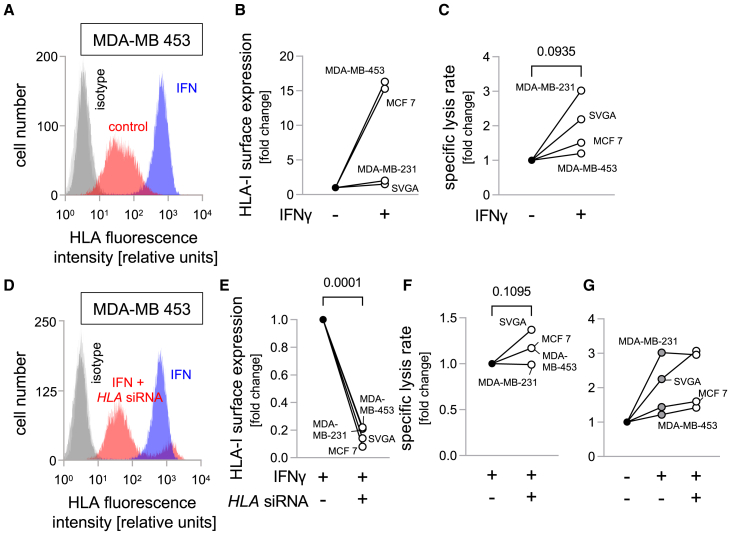
IFNγ upregulates HLA-I expression on target cell lines, but, nevertheless, increases cytotoxicity of HER2-specific CAR NK-92 cells (A) Representative histograms of HLA-I-associated fluorescence intensity and respective IgG isotype controls of MDA-MB-453 cells incubated with IFNγ (blue and dark gray) or without IFNγ (red and light gray). (B and C) IFNγ incubation upregulates mean (n = 2–3 per cell line) HLA-I surface expression normalized to vehicle control (B) and increases specific lysis (C) by HER2-directed CAR NK-92 cells (E:T ratio 2.5:1) for MDA-MB-453, MDA-MB-231, MCF7, and SVGA cells. (D) Representative histograms of HLA-I-associated fluorescence intensity and respective IgG isotype controls of MDA-MB-453 target cells after IFNγ incubation (blue and dark gray), or IFNγ incubation with additional HLA-I directed siRNA treatment (red and light gray). (E–G) HLA-I downregulation by siRNA during IFNγ incubation reduced mean (n = 2–3 per cell line) HLA-I surface expression (E) and increased relative (F) and absolute (G) specific lysis by HER2-directed CAR NK-92 cells (E:T ratio 2.5:1) for MDA-MB-453, MCF7 and SVGA, but not MDA-MB-231 cells. Numbers indicate *p* values as calculated by paired t-test. Absolute values for each experiment are shown in Figure S12.

There is ample evidence of HLA-I upregulation in cancer cells by IFNγ,^38^ which could potentially interfere with CAR NK-92 cell activity. Hence, we incubated breast cancer cell lines (MDA-MB-453, MDA-MB-231 and MCF7) as well as non-malignant SVGA cells with IFNγ for 48 h. This resulted in an increase in HLA-I surface expression in all four cell lines, with an on average 15-fold increase in MDA-MB-453 and MCF7 cells with low basal HLA-I expression. A 2-fold increase in HLA-I expression was found in HLA-I^intermediate^ MDA-MB-231 cells, and a more moderate 1.5-fold increase in HLA-I^high^ SVGA cells (Figures 6A and 6B). Unexpectedly, IFNγ pre-treatment did not lead to reduced lysis by the CAR-NK cells, but instead resulted in a consistent increase in respective lysis rates for all four cell lines (Figure 6C). To further evaluate the importance of HLA-I expression for cell killing, we co-applied IFNγ and *HLA-I* siRNA to the target cells. Unlike IFNγ treatment alone, this co-treatment prevented an increase in HLA-I surface expression (Figures 6D and 6E), with the HLA-I level comparable to the endogenous level in the absence of IFNγ and *HLA-I-*siRNA (Figures 6A and 6D). The co-treatment increased lysis by CAR-NK cells moderately for SVGA cells, and marginally for MCF7 and MDA-MB-453 cells (Figure 6F). Lysis rates were not affected after additional *HLA-I* siRNA treatment in the case of MDA-MB-231 cells, potentially due to the pronounced effect of IFNγ alone. Overall, both pre-treatment of target cells with IFNγ alone, or IFNγ and *HLA-I* siRNA led to increased lysis rates for all four cell lines tested (Figure 6G).

## Discussion

In our study, we investigated the influence of target antigen density and HLA-I expression on target cell killing by CAR-engineered NK cells. Thereby, we observed only a small dependence of the activity of CD276-directed CAR NK-92 cells on target antigen density. Accordingly, irradiation-induced upregulation of CD276 in glioblastoma cells did not lead to enhanced killing by the CD276-specific CAR-NK cells. In contrast, HER2-directed CAR NK-92 cells showed a pronounced dependence on target antigen density. Previous studies found that the killing efficiency of CAR T cells does not only depend on the specific tumor antigen but especially on its expression density on cancer cells.^39^^,^^40^ Accordingly, a common escape mechanism of cancer cells to CAR T cell therapy is the downregulation of the target antigen.^41^ Other studies have shown that certain CAR designs have different minimum antigen level requirements for full activation.^42^^,^^43^ As the CAR constructs in both CAR NK-92 cell lines, with the exception of the scFv cell targeting domains and promotors used, are very similar, the different dependence on target antigen density must have other causes. Accordingly, different affinities of the single chain fragment, variable antibody domains for their targets, different expression levels of the CARs, or specific features of the targeted antigens may have contributed. On the other hand, we found similar activation thresholds for both CAR NK-92 cell lines. Interestingly, other studies have reported antigen-independent tonic signaling when using a CD276-specific CAR construct,^44^ a well-established driver of CAR-T cell exhaustion.^45^ While strong activation of CAR-NK cells by low levels of target antigen may be beneficial to eradicate tumor cells in the short term, this may increase the risk of on-target/off-tumor toxicities and, similar to tonic signaling, may lead to early CAR-NK cell exhaustion. Hence, it may be beneficial to focus on CAR-NK clones with a more pronounced dependence on target antigen density.

Prompted by the weak dependence on target antigen density of the CD276-directed CAR NK-92 cells, we next studied potential on-target/off-tumor effects. In contrast to previous findings,^25^^,^^33^ non-malignant cell lines expressed CD276 to a similar extent as the tested pGSCs in our study. Nevertheless, lysis rates after co-incubation with CAR-NK cells were less than half of those observed for cancer cells. The findings from our subsequent experiments indicate that this was driven by the more pronounced HLA-I expression of the non-malignant cells. HER2-directed CAR NK-92 cells showed a less pronounced modulation by HLA-I expression of target cells, which was only evident in the case of SVGA (but not hCMEC/D3) cells. One might speculate that these differences were due to the lower expression of the inhibitory receptors NKG2A and ILT-2 by HER2-directed CAR NK-92 cells when compared to CD276-specific CAR NK-92. Overall, these findings suggest that the activity of CAR NK-92 cells is still modulated by the HLA-I expression of target cells, albeit to a different extent, depending on the effector cell type analyzed. Modulation by HLA-I levels on target cells has been described previously in other studies for CAR-NK cells derived from peripheral blood mononuclear cells^8^ or induced pluripotent stem cells.^9^ Since NK-92 cells display an activated NK-cell phenotype while expressing only few of the inhibitory NK-cell receptors such as KIRs and NKG2A,^16^^,^^23^ their ability to differentiate between cancer and normal cells carrying the CAR target antigen may be impaired to some extent. Hence, focusing on CAR NK-92 cell clones with relatively high levels of inhibitory receptors may reduce on-target/off-tumor-toxicity.

In general, this may be a preferred feature when translating CAR-engineered cells to solid tumors, as tumor-specific antigens are rare, and on-target/off-tumor toxicity presents a potentially serious risk to the development of CAR-T and CAR-NK cell products.^6^ While no severe side effects or dose-limiting toxicities were reported upon treatment of AML and glioblastoma patients with CD33^−^and HER2-specific CAR NK-92 cells in early phase clinical trials,^11^^,^^12^ this may be different in future studies depending on the chosen CAR target antigen, effector cell dose or duration of treatment. Different approaches have been proposed to circumvent or lower on-target/off-tumor toxicity, including combinatorial CAR constructs,^46^ SynNotch receptors,^47^ or logic-gated intracellular network CAR constructs.^44^ All these approaches make CAR effector cell activation dependent on the (simultaneous) presence of two different target antigens, which may alleviate toxicity. However, this may also interfere with the efficacy of CAR-T and CAR-NK cells, or facilitate tumor immune evasion by antigen loss.^48^ Another strategy is to transduce cells with an additional inhibitory CAR construct (iCAR) or other inhibitory receptors, which inhibit activation of effector cells after recognition of the respective antigens or receptor ligands on normal cells.^49^ In essence, this approach recapitulates the intrinsic regulation of NK cell activity by integrating signals from endogenously expressed activating and inhibitory receptors. Among the few direct comparisons of CAR-T and CAR-NK cells available, one study indeed reported less toxicity and better retained efficacy of mesothelin-directed CAR-iPSC-NK cells when compared to CAR-T cells in *in vivo* models.^50^

Lastly, our findings of enhanced target cell lysis after IFNγ pre-treatment (albeit an increase in HLA-I surface expression) indicate that additional factors other than HLA-I expression on target cells can further influence CAR NK-92 cell activity. Other studies reported an induction of ICAM-1 after IFNγ incubation, facilitating target cell lysis.^51^^,^^52^

In conclusion, we report differences in the dependence of two lines of CAR NK-92 cells on target antigen density and their modulation by HLA-I expression on target cells. These findings may become relevant to select specific CAR-NK cell clones or CAR constructs to fine-tune anti-cancer efficacy while simultaneously limiting the risk of on-target/off-tumor toxicity.

### Limitations of the study

It was not possible to delineate the mechanisms underlying the different dependence of CD276-and HER2-targeted CAR NK-92 cells on target antigen density. Second, while SVGA and hCMEC/D3 cells are commonly used as non-malignant cell lines, both cell types are immortalized, and may not fully reflect “normal” cells from healthy tissues. Third, we utilized non-irradiated NK-92 cells for our experiments. Previous work with both CAR NK-92 cell lines showed that they transiently retain full activity after irradiation, even under immunosuppressive or hypoxic conditions.^19^^,^^20^ Furthermore, irradiation did not significantly affect their cytokine profile or the expression of activating NK cell receptors.^53^ Our own data indicate a retained (CD276-CAR NK-92 cells) or even increased expression of inhibitory receptors (NKG2A in HER2-CAR NK-92 cells), suggesting a retained or (potentially) higher selectivity, respectively. Lastly, in our present study we only analyzed the CAR NK-92 cells in *in vitro* experiments. Follow-up work in suitable animal models will be required to investigate whether the observed differences in lysis rates between non-malignant and cancer cell lines as targets are also observed *in vivo*. Still, our present study and the work of others^8^^,^^9^ suggest that inhibitory receptors on CAR-NK cells may indeed enable the fine-tuning of anti-tumor activity. Last, our work focused on CAR NK cells and, hence, we cannot draw conclusions about similar effects in CAR T cells. Theoretically, a sustained killing of normal cell lines with retained HLA-I expression is expected as only a small subset of T cells express HLA-I -specific inhibitory receptors.^54^^,^^55^^,^^56^ Similarly, our experiments cannot dissect whether the differences in the dependence of the target antigen density of both CAR constructs is transferable to CAR T cells.

## Resource availability

### Lead contact

Further information and requests for resources and reagents should be directed to and will be fulfilled by the lead contact, Nicolai Stransky (nicolai.stransky@med.uni-tuebingen.de).

### Materials availability

This study did not generate new unique reagents.

### Data and code availability

Data is available upon reasonable request from the corresponding author.

## Acknowledgments

The authors thank Heidrun Faltin for excellent technical assistance. CD276-specific CAR NK-92 cells were kindly provided by Sabine Schleicher (Department for Pediatrics, University Hospital Tübingen), HFFs by Evi Schmid (Department of Pediatric Surgery and Pediatric Urology, University Children’s Hospital Tübingen) and MDA-MB-453 cells by Mahmoud Toulany (Department of Radiation Oncology, University Hospital Tübingen). This work was supported by the 10.13039/501100005972German Cancer Aid grant numbers 70112872 and 70113144; Gesellschaft für Kinderkrebsforschung; 10.13039/501100001659German Research Foundation Grants DFG EC 575/2-1 and HU 781/7-1; and of the IZKF Medical Faculty Tübingen. The graphical abstract was created using https://BioRender.com.

## Author contributions

Conceptualization, N.S., S.M.H., and F.E.; methodology, N.S., S.M.H., and F.E.; investigation, N.S., R.J., L.P., and K.G.; data curation, N.S., R.J., L.P., and S.M.H.; writing – original draft, N.S., R.J., and S.M.H.; writing – review and editing: all authors; funding acquisition, F.E., S.M.H., and P.R.; resources, W.S.W., S.M.H., P.R., and F.E.; supervision S.M.H., P.R., and F.E.

## Declaration of interests

W.S.W. is named as an inventor on patents owned by Georg-Speyer-Haus that relate to HER2-targeted CAR-NK cells. The authors declare no other potential conflicts of interest.

## STAR★Methods

### Key resources table

**Table:** 

REAGENT or RESOURCE	SOURCE	IDENTIFIER
**Antibodies**		

FITC-labelled anti-CD276 antibody	Miltenyi Biotec	#130-124-217
FITC-labelled isotype control	Miltenyi Biotec	#130-123-667
FITC-labelled anti-HLA-I antibody	BioLegend	#311404
FITC-labelled isotype control	BioLegend	#400208
PE-labelled anti-HER2 antibody	Miltenyi Biotec	#130-124-466
PE-labelled anti-NKG2A antibody	Miltenyi Biotec	#130-114-092
PE-labelled anti-ILT2 antibody	Miltenyi Biotec	#130-116-736
PE-labelled anti-KIR2DL4 antibody	Miltenyi Biotec	#130-112-537
PE-labelled anti-KIR2DL1 antibody	Miltenyi Biotec	#130-120-586
PE-labelled anti-NKp30 antibody	Miltenyi Biotec	#130-112-502
PE-labelled anti-NKG2D antibody	Miltenyi Biotec	#130-111-723
PE-labelled isotype control	Miltenyi Biotec	#130-113-438

**Chemicals, peptides, and recombinant proteins**		

Recombinant Human IL-2	R & D Systems	#202-IL-050/CF
Recombinant Human IFNγ	R&D Systems	#10067-IF-025
Biotinylated Human Her2 protein	AcroBioscience	HE2-H82E2-25ug
Biotinylated Human B7-H3 protein	AcroBioscience	B73-H82F5-25ug
Lipofexatmin RNAiMAX	Thermo Fisher	#13778075
calcein acetoxymethyl ester	Thermo Fisher	#C3100MP
green fluorescent probes	Invitrogen	#C2925
Propidium iodide solution	Sigma Aldrich	P4864

**Critical commercial assays**		

DuoSet ELISA Ancillary Reagent Kit 2	R & D Systems	#DY008 or #DY008B
Human TNF-alpha DuoSet ELISA	R & D Systems	#DY210-05
Human IFN-gamma DuoSet ELISA	R & D Systems	#DY285B-05

**Experimental models: Cell lines**		

AR6	Isolated from patient (Ganser et al.^57^)	N/A
LK7	Isolated from patient (Ganser et al.^57^)	N/A
LK11	Isolated from patient (Ganser et al.^57^)	N/A
LK17	Isolated from patient (Ganser et al.^57^)	N/A
LK31	Isolated from patient (Ganser et al.^57^)	N/A
Jurkat j16	ATCC	RRID:CVCL_0065
K562	ATCC	RRID:CVCL_0004
MDA-MB-231	Laboratory of Mahmoud Toulany	RRID: CVCL_0062
MDA-MB-453	Laboratory of Mahmoud Toulany	RRID:CVCL_0418
MCF-7	Laboratory of Mahmoud Toulany	RRID:CVCL_0031
U-87MG	ATCC	RRID:CVCL_0022
SVGA	Laboratory of Walter J. Atwood	RRID:CVCL_5G13
hCMEC/D3	Laboratory of Walter J. Atwood	RRID:CVCL_U985
HFF	Laboratory of Evi Schmid	N/A
NK-92	Laboratory of Sabine Schleicher	RRID:CVCL_2142
CD276-specific CAR NK-92	Reported previously (Grote et al.^31^)	N/A
HER2-specific CAR NK-92	Reported previously (Schönfeld et al.^32^)	N/A

**Oligonucleotides**		

Anti-*CD276* siRNA	Horizon discovery	#L-007813-01-0005
Anti-*B2M* siRNA	Horizon discovery	#L-004366-00-0005
Anti-*HER2* siRNA	Horizon discovery	#L-003126-00-0005
Non-targeting siRNA	Horizon discovery	#13778075

**Software and algorithms**		

FCSExpress-3 software	*De Novo* Software	
GraphPad Prism (version 9.4.1)	Graphpad Software Inc.	

### Experimental model and study participant details

All sections are written in accordance with MeRIT (Method Reporting with Initials for Transparency).^58^

*Cell culture* pGSCs (AR6, LK7, LK11, LK17, LK31) were grown from resectates as described previously^57^ and propagated in complete human NeuroCult NS-A Proliferation Medium (STEMCELL Technologies, #05751, #78003, #78006.2, #07980). Hematological cancer cell lines (Jurkat j16, K562) were grown in RPMI medium (Thermo Fisher, #21875-034) containing 10% fetal bovine serum (FBS). Non-neoplastically transformed normal cell lines (SVGA, hCMEC/D3, HFF) as well as breast cancer or glioma cell lines (MDA-MB-231, MDA-MB-453, MCF-7, U-87MG) were propagated in DMEM medium (Thermo Fisher, #41965-039) containing 10% FBS. Effector cell lines (NK-92, CD276-specific CAR NK-92, HER2-specific CAR NK-92 cells) were described previously^31^^,^^32^ and grown in MEMα medium (Thermo Fisher, #32561-029) containing 20% FBS and 100 IU/mL IL-2 (R & D Systems, #202-IL-050/CF), or X-VIVO 10 Serum-free Hematopoietic Cell Medium (Lonza Biozyme, #BE04-380Q) containing 5% human serum (Bio&Sell, #OTCAB.SE.0100) and 100 IU/mL IL-2. ErbB2-specific monoclonal antibody FRP5 has an apparent K_D_ of 0.82 nM, while the apparent affinity of recombinant scFv(FRP5) protein has been reported as 7.2 nM.^59^ Thereby, scFv(FRP5) recognizes a discontinuous epitope within N-terminal domain I of human ErbB2/HER2.^60^ According to the manufacturer, the binding affinity of Clone CD276.6 (m856) to human CD276 over time (s) as measured by surface plasmon resonance is D = 1.3 x 10^9^ M. All cells were propagated at 37°C and 10% (DMEM) or 5% CO_2_ (all other media). Cells were dissociated enzymatically with Accutase (Sigma-Aldrich, #A6964) for 5-10 min and cell numbers were determined using Hemocytometer Chips. Cell lines have not been authenticated specifically for this study or tested routinely for mycoplasm contamination.

### Method details

#### Flow cytometry

Cell staining procedures for flow cytometry experiments were conducted as detailed in the manufacturer’s instructions by NS, RJ or LP. After dissociation and one-time washing with phosphate-buffered saline, cells were stained with respective antibodies: anti-CD276 (1:50; Miltenyi Biotec; #130-124-217; isotype control: #130-123-667), anti-HLA-I (1:20; BioLegend; #311404; isotype control: #400208), anti-HER2, anti-NKG2A, anti-ILT-2, anti-KIR2DL4, anti-KIR2DL1, anti-NKp30 and anti-NKG2D (all 1:50; Miltenyi Biotec; #130-124-466; #130-114-092; #130-116-736; #130-112-537; #130-120-586, #130-112-502, #130-111-723 isotype control: #130-113-438). Samples were incubated for 10-15 minutes in darkness, after which unbound antibody was washed off. All flow cytometry experiments were conducted on a FACSCalibur cytometer (BD) and analyzed by FCSExpress-3 software (version 3.00.0825, *De Novo* Software). Fluorescence intensities reported are geometric mean values of each antibody corrected for the geometric mean values of respective isotype control.

#### Fluorescence microscopy

To visualize the effector function of CAR NK-92 cells, spheroids of GFP-transduced LK17 cells were pipetted into 96 well plates, followed by addition of CD276-specific CAR NK-92 cells or NK-92 cells. Microphotographs were taken by LP at reported time points after the addition of effector cells on an Axiovert 25 inverse microscope (Zeiss) and an EOS 500D digital camera (Canon).

#### Cytotoxicity assay

Target cells were stained with 9 μmol/L calcein acetoxymethyl ester (Calcein-AM; Thermo Fisher, #C3100MP) at a final density of 1x10^5^ cells/mL for 50 min. After removing remaining calcein-AM by serial centrifugation and discarding the supernatant, 5000 target cells were co-incubated with (CAR) NK-92 cells at different effector-to-target (E:T) cell ratios in 200 μL for 2 hours. After centrifuging the samples at 250 g for 5 min, 100 μL of the supernatant was used to determine calcein release by measuring the fluorescence with a Spark multimode microplate reader (Tecan). Spontaneous calcein release was measured using target cells only, while target cells were incubated with 2% Triton-X100 to determine quantitative calcein release. Medium used was RPMI containing 2% FBS. Specific lysis rates were calculated as follows:

$$specific\;lysis=\frac{FI\;\left(sample\right)-FI\;\left(spontaneous\;release\right)}{FI\;\left(triton\;release\right)-FI\;\left(spontaneous\;release\right)}$$

(withFl=fluorescenceintensity)

For flow cytometry based killing assays, target cells were stained with 10 μmol/L green fluorescent probes (Invitrogen, #C2925) for 30 minutes and subsequently co-incubated with the effector cells for a total of 24 hours in RPMI containing 2% FBS. Afterwards, all cells were stained with 10 μg/mL of propidium iodide for 20 minutes. Dead target cells were defined by positive double staining. Experiments were performed by NS or LP (CD276-specific CAR NK-92) or RJ with the help of NS (HER2-specific CAR NK-92).

#### Knockdown experiments

siRNA oligonucleotides targeting *CD276*, *B2M*, *HER2* and a non-targeting control (all horizon discovery; #L-007813-01-0005, #L-004366-00-0005, #L-003126-00-0005, #D-001810-10-05) were transfected with Lipofectamine RNAiMAX (Thermo Fisher, #13778075) according to the manufacturer’s “RNAiMAX Reverse Transfections Lipofectamine” instruction by NS or RJ. Concentrations and incubation periods were: *CD276* siRNA: 20 nmol/L and 72 hours; *B2M* siRNA: 10 or 20 nmol/L and 48 or 72 hours (depending on cell line); non-targeting control: accordingly). The efficacy of siRNA knockdown was assessed by flow cytometry.

#### Radiation treatment

Radiation treatment was applied by NS or LP using a 6 MV linear accelerator (LINAC SL25 Philips) at a dose rate of 5 Gy/min at room temperature.

#### IFNγ incubation

For IFNγ treatment, around 600,000-900,000 target cells (depending on cell line) were incubated with Recombinant Human IFNγ (R&D Systems, #10067-IF-025) for 48 or 72 hours using an IFNγ concentration of 100 ng/ml and respective volumes of PBS as negative control. Simultaneously, knockdown experiments were carried out with siRNA targeting HLA-I and a non-targeting control, and the resulting HLA-I up- or downregulation was monitored by flow cytometry. Experiments were performed by RJ.

#### Enzyme-linked immunosorbent assay (ELISA)

Target cells were incubated with effector cells in RPMI medium containing 2% FCS for 5 hours at a cell density of 666,667 cells/mL (CD276-directed CAR NK-92 cells) or 333,333 cells/mL (HER2-directed CAR NK-92 cells) and an effector-to-target ratio of 1:1. Afterwards, samples were centrifuged at 250 g for 5 min and the supernatant was collected. Cytokine release was quantified with enzyme-linked immunosorbent assay kits (all R & D Systems; DuoSet ELISA Ancillary Reagent Kit 2, #DY008 or #DY008B; Human TNF-alpha DuoSet ELISA, #DY210-05; Human IFN-gamma DuoSet ELISA, #DY285B-05) according to the manufacturer’s instructions. Optical density was measured on an Anthos 2010 microplate reader (Anthos Mikrosysteme GmbH). Experiments were performed by NS or RJ.

#### Activation threshold

To define activation thresholds of the CAR NK-92 cells, we used Biotinylated Human B7-H3 / CD276 Protein, Biotinylated Human Her2 / ErbB2 Protein and Streptavidin Coated Plates (all AcroBioscience; B73-H82F5-25ug; HE2-H82E2-25ug; SP-11-1plate) according to the manufacturer’s instruction. In short, proteins were loaded onto the plates at 37°C for 1 hour. After washing off unbound protein, the effector cells were pipetted into the wells and incubated in RPMI medium containing 2% FCS for 5 hours at a cell density of 400.000 cells/mL. After centrifuging the samples at 250 g for 5 min, the supernatant was collected and analyzed for TNF-alpha and IFN-gamma by ELISA. All experiments were performed by NS.

### Quantification and statistical analysis

Data visualization and statistical tests were performed using GraphPad Prism (version 9.4.1) by NS, RJ and SMH. In general, visualized data points are from independent experiments, and statistical tests were performed using values of independent experiments or average values per cell line. Specifics are given in the respective figure legend.
