# Supplementary material for: Chimeric antigen receptor NK-92 cell function is modulated by HLA class I expression of target cells
Source: iScience. 2025 Apr 23;28(5):112523. doi: 10.1016/j.isci.2025.112523 (PMC12135394; doi:10.1016/j.isci.2025.112523)

iScience, Volume *28*

Supplemental information

Chimeric antigen receptor NK-92 cell function is modulated by HLA class I expression of target cells

Nicolai Stransky, Ranran Ji, Lukas Prause, Katrin Ganser, Winfried S. Wels, Peter Ruth, Stephan M. Huber, and Franziska Eckert

Figure S1: Parental NK-92 only lyse K562 and Jurkat j16 to a low degree, but display no detectable activity against pGSCs or non-malignant cell lines.

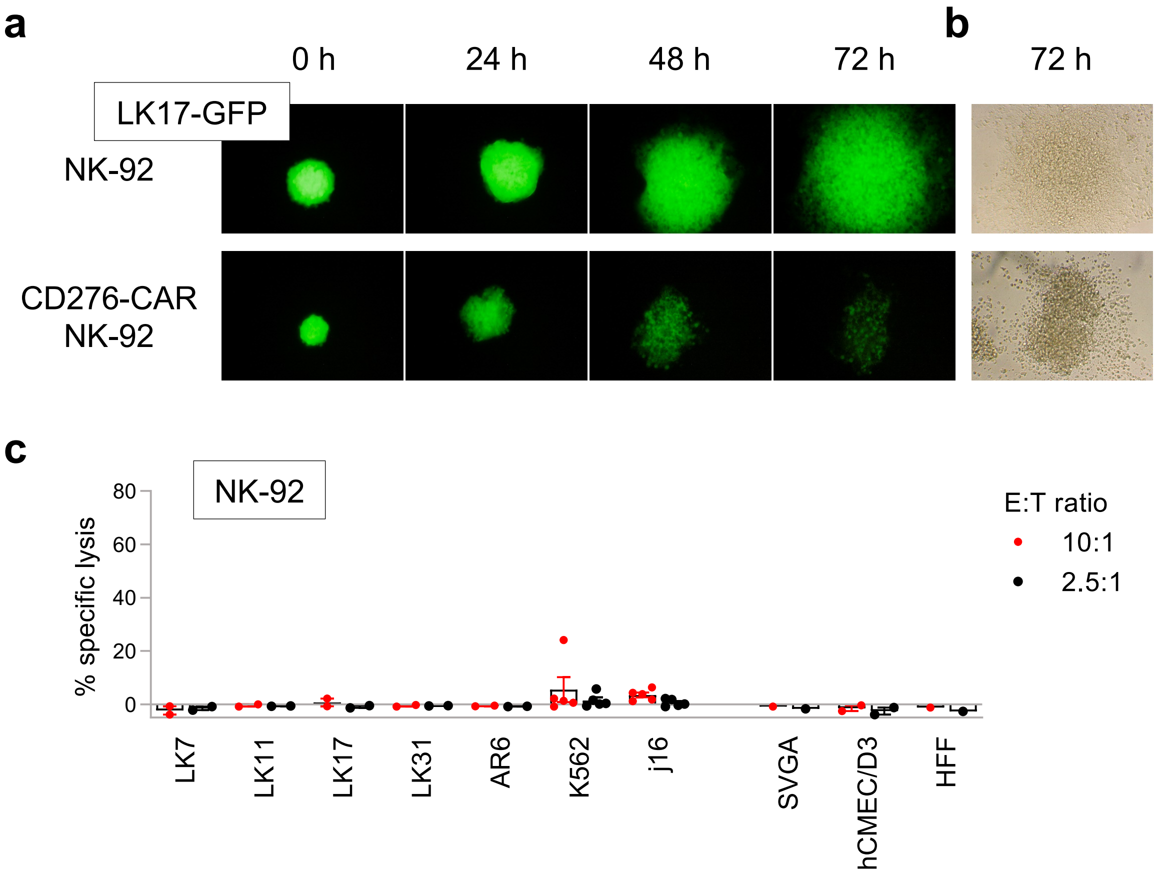

Figure S2: K562 cells express little and Jurkat j16 cells no CD276 on their surface.

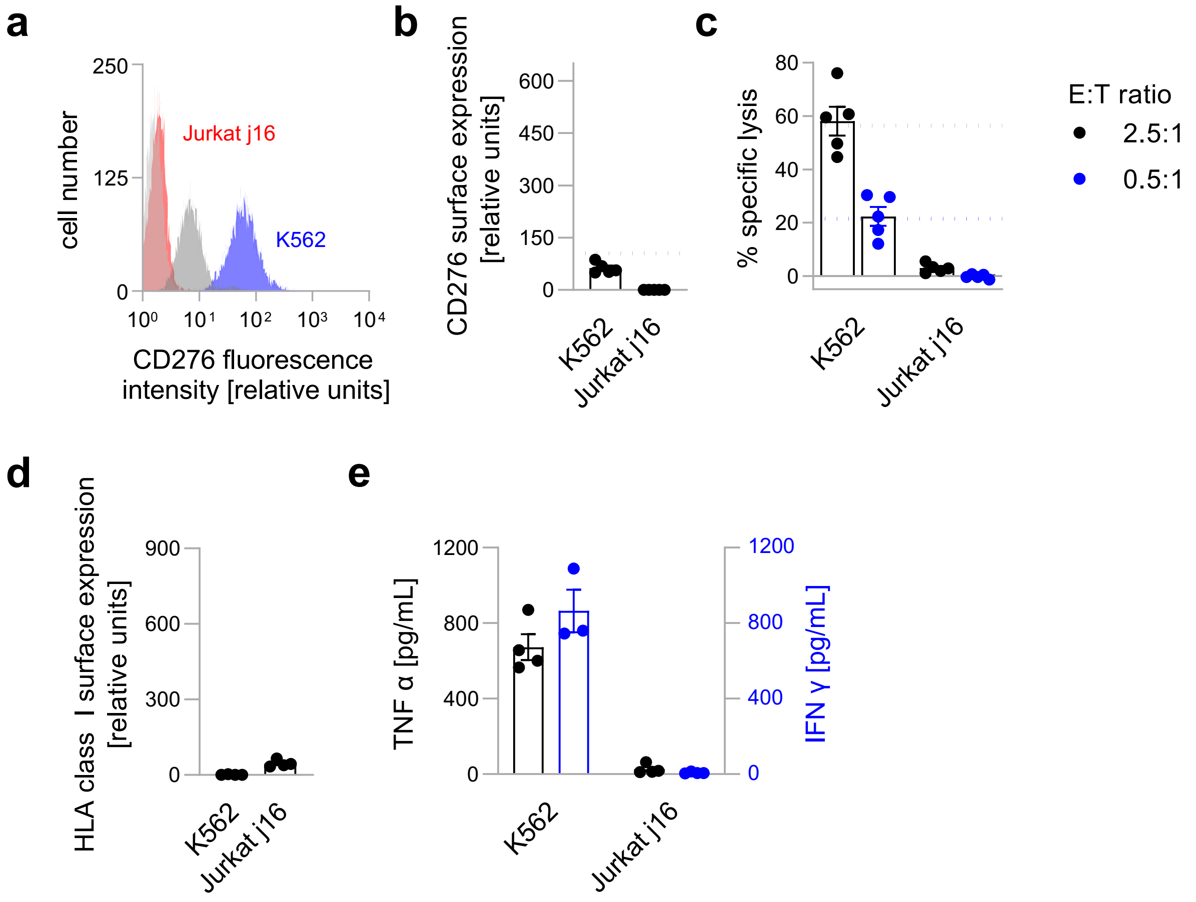

Figure S3: Irradiation increases CD276 surface expression in LK7, LK11 and LK17 glioblastoma cells, but does not meaningfully impact lysis rates after co-incubation with CD276-directed CAR NK-92 cells.

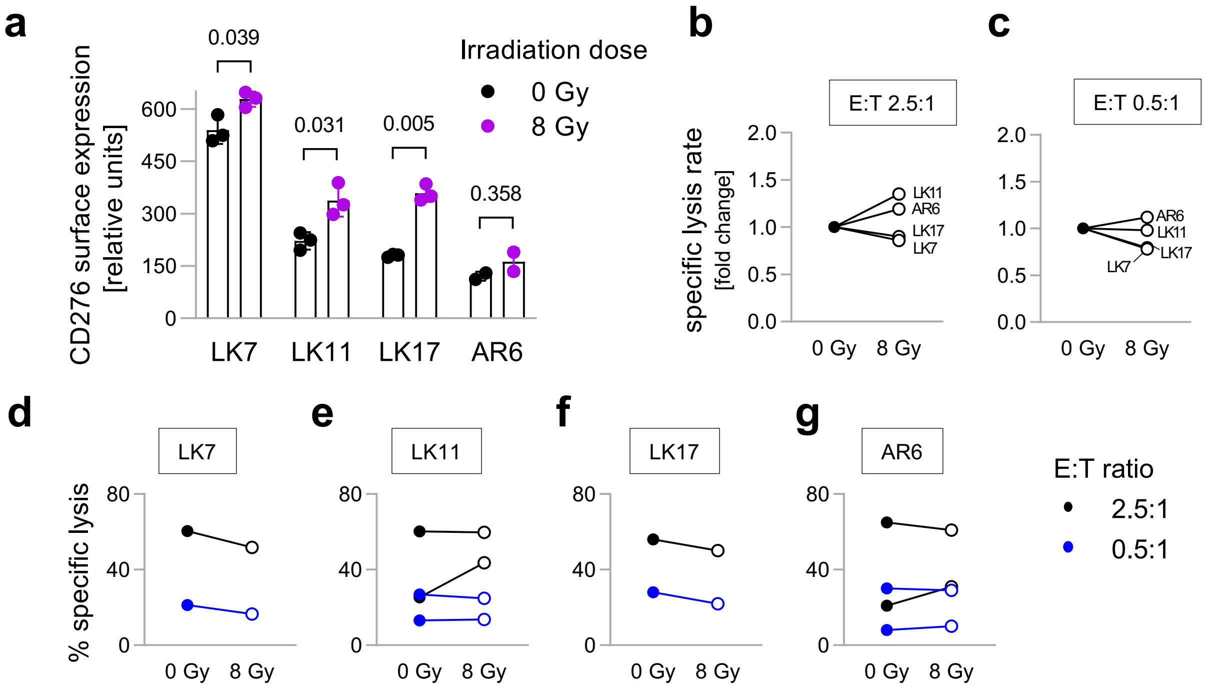

Figure S4: Downregulation of CD276 by siRNA in target cells consistently reduces lysis rates after co-incubation with CD276-directed CAR NK-92 cells, although only to a small extent.

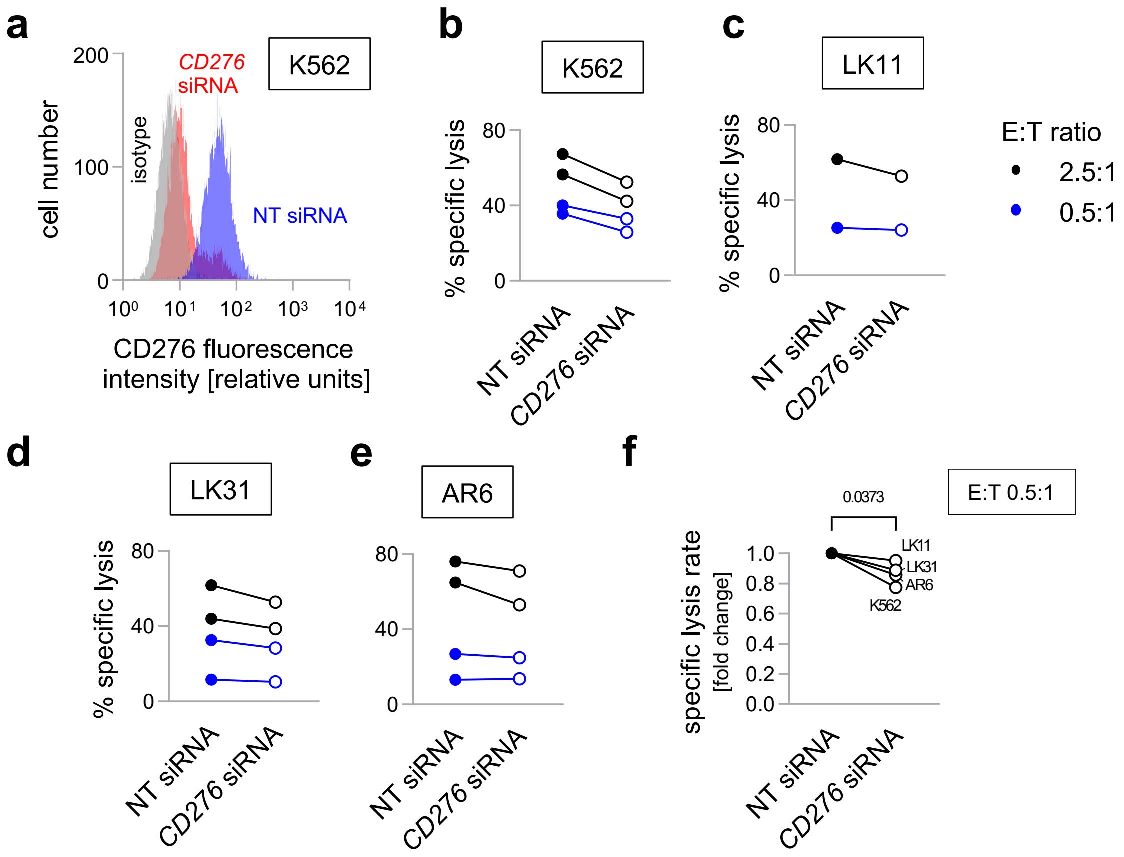

Figure S5: Flow cytometry based killing assay of CD276-CAR NK-92 cells.

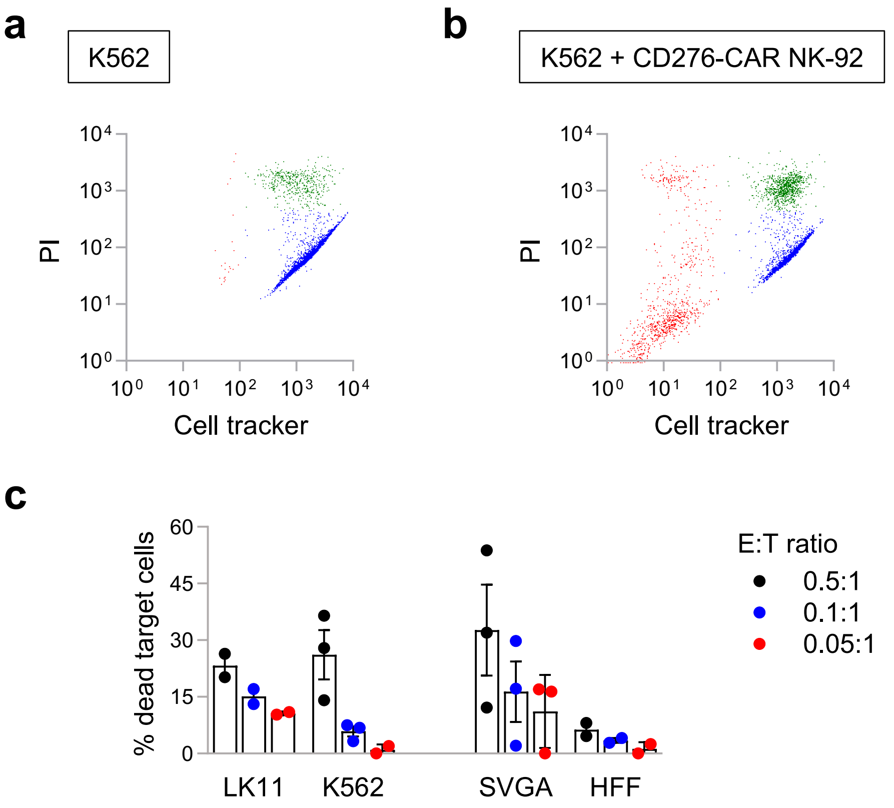

Figure S6: HLA-directed siRNA leads to increased killing of HLA-I^{high} cells by CD276-directed CAR NK-92 cells, compared to no changes in the killing of HLA-I^{low} cells.

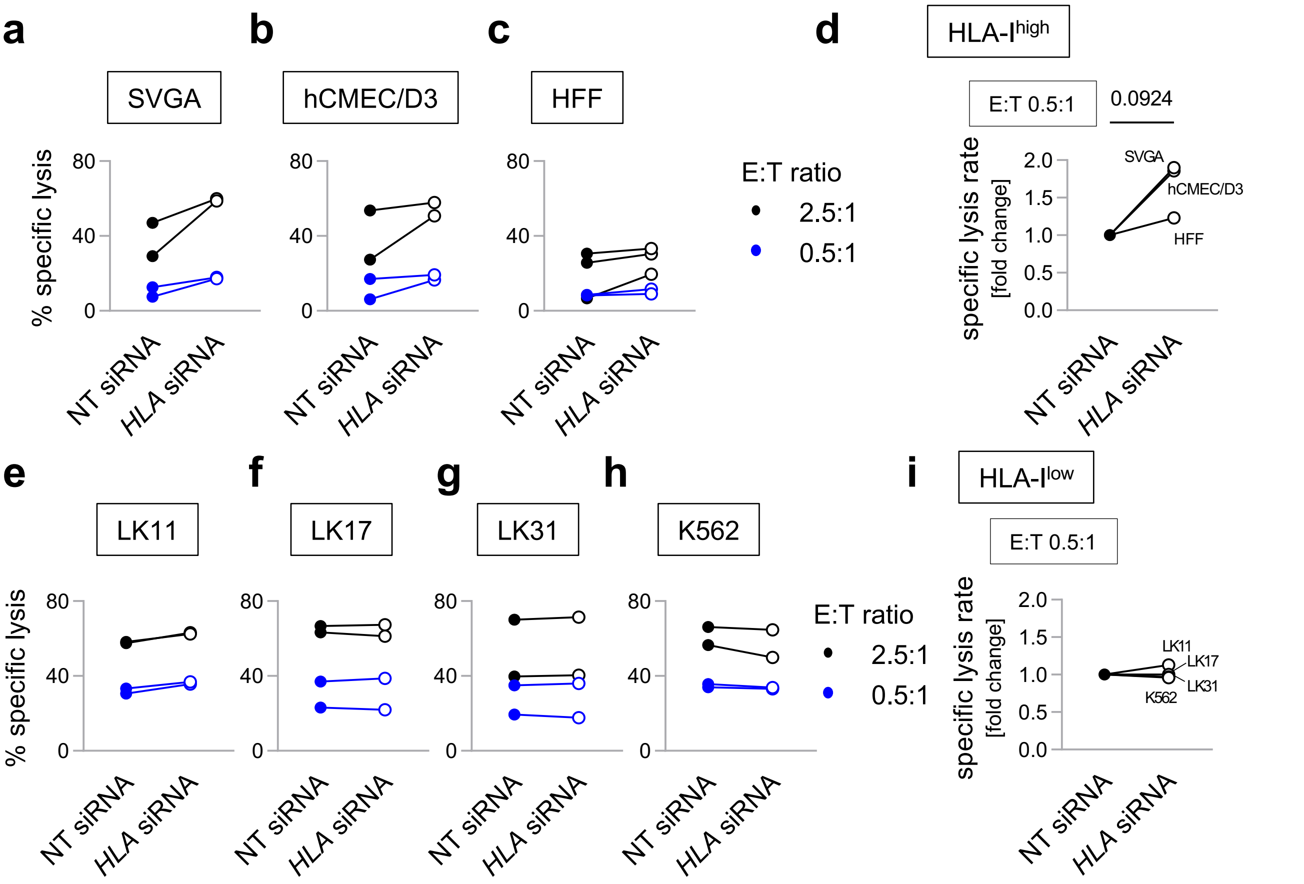

Figure S7: HLA-I expression of target cells modulates secretion of TNFα and IFNγ by CD276-specific CAR NK-92 cells.

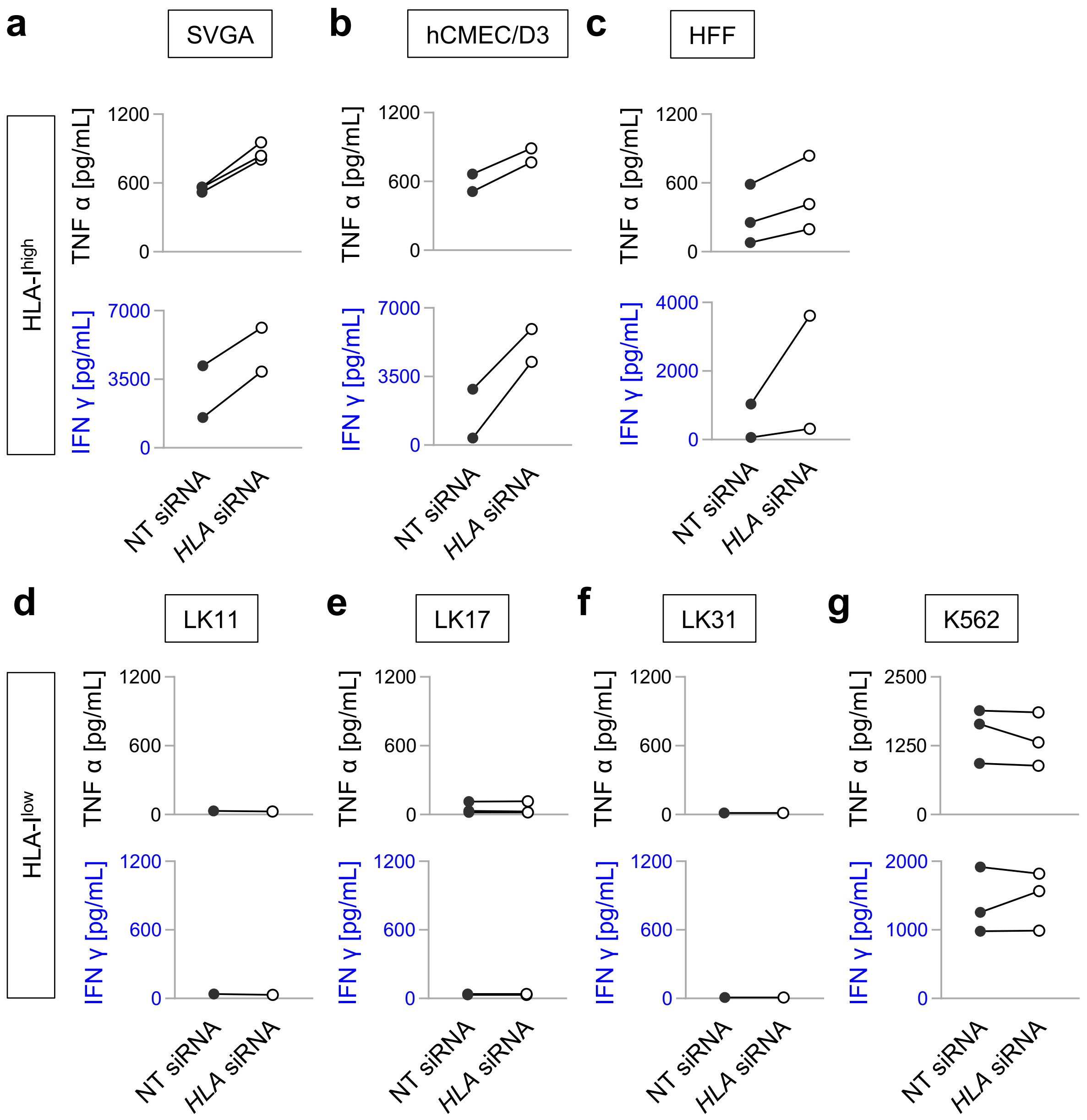

Figure S8: pGSCs express little (LK7, LK11) or no HER2 (LK17, LK31, AR6) on their surface.

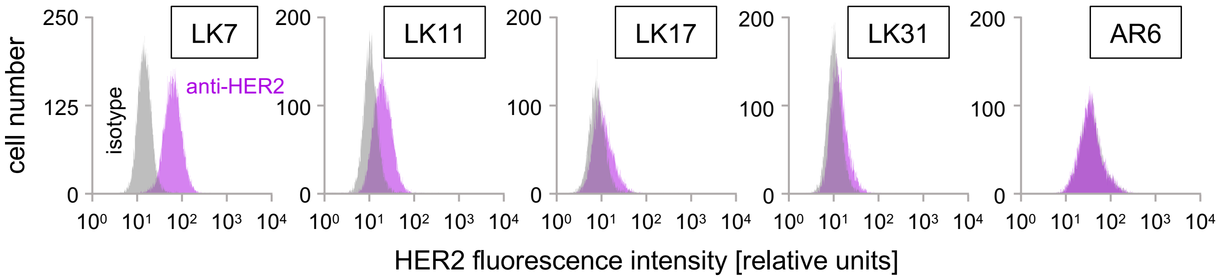

Figure S9: Activation threshold of CAR NK-92 cells.

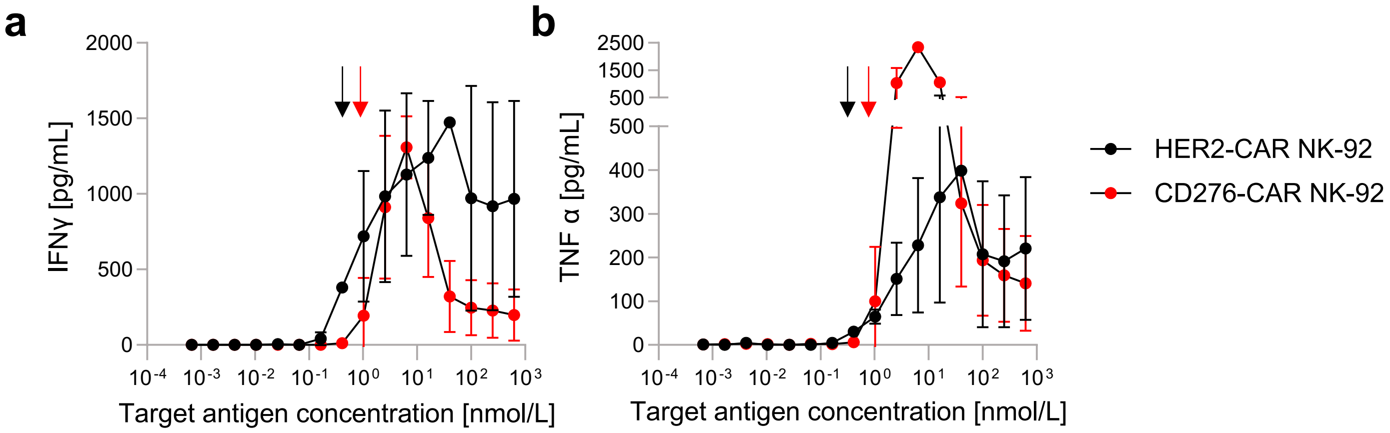

Figure S10: HER2-directed CAR NK-92 cells express less ILT-2 and NKG2A than CD276-directed CAR NK-92 cells.

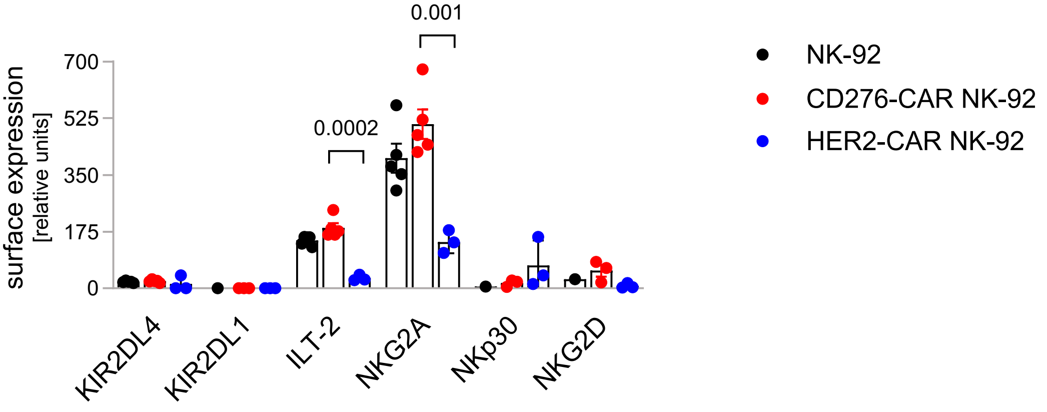

Figure S11: Influence of Irradiation on surface expression of activating/inhibitory receptors of CAR NK-92 cells.

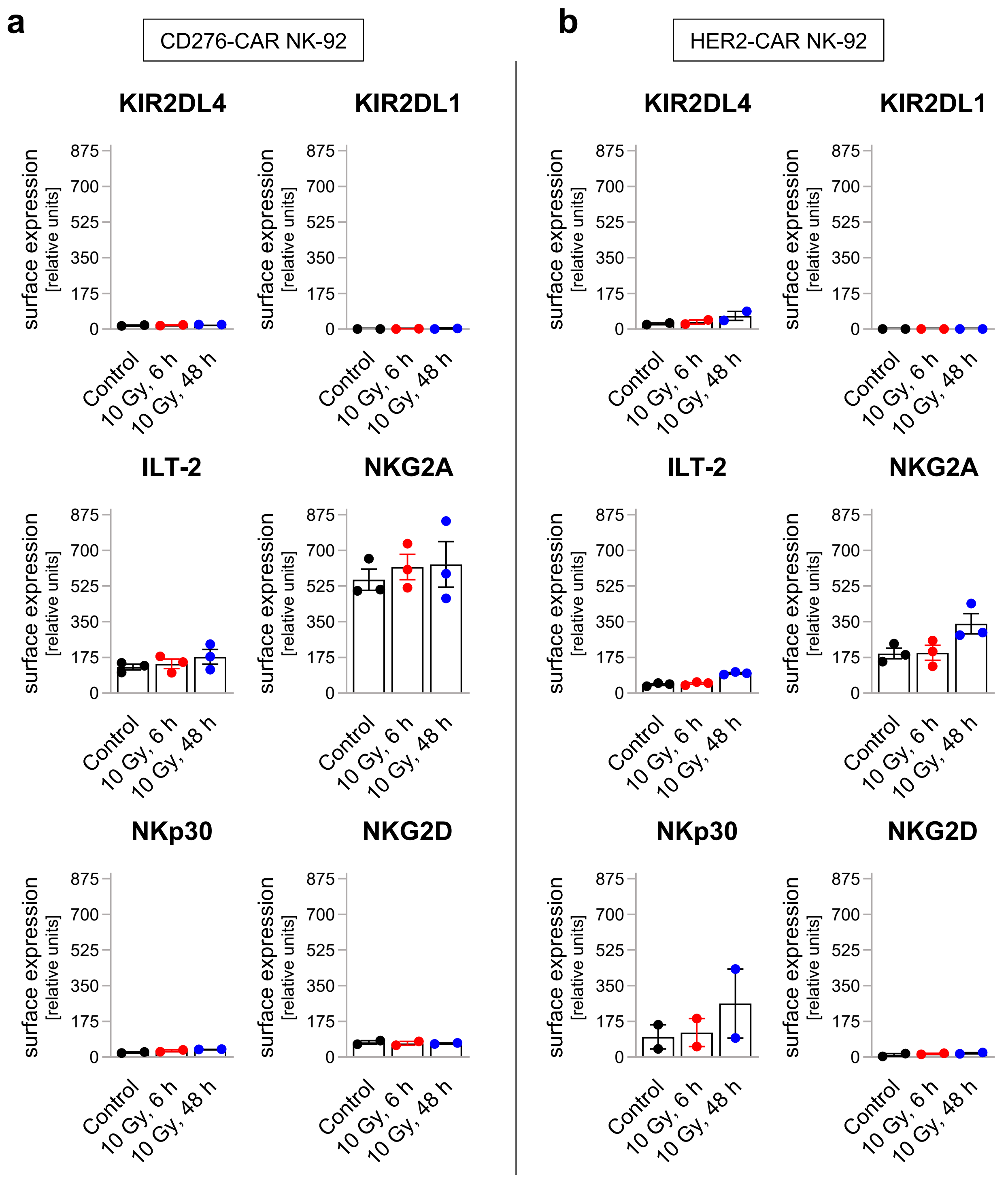

Figure S12: Pre-incubation of target cells with IFNγ increases HLA-I expression but does not lead to impaired HER2-directed CAR NK-92 cell killing.

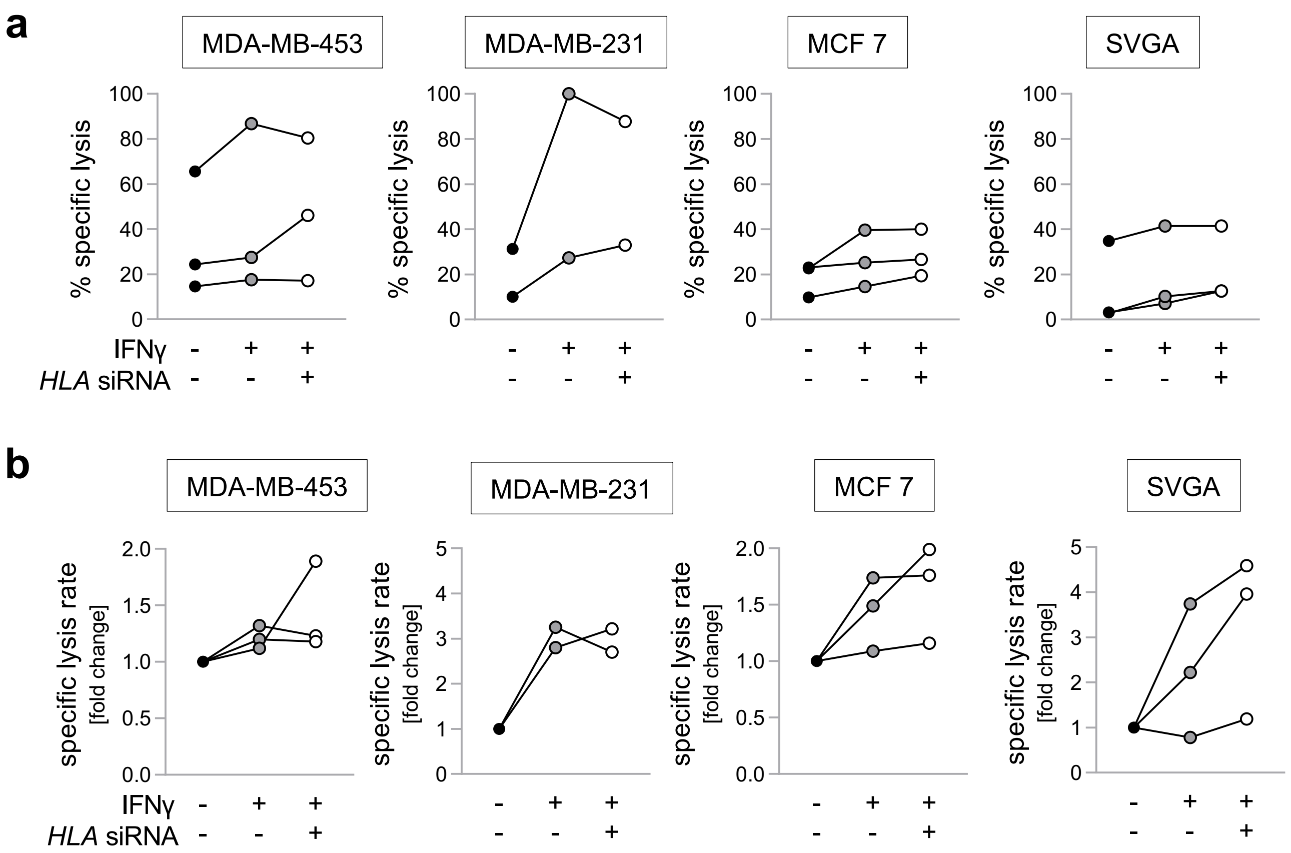

Supplement: Document S1. Figures S1–S12 [file mmc1.pdf]
